# Baseline lncRNA PCAT1 high expression and its longitude increment during induction therapy predict worse prognosis in multiple myeloma patients

**DOI:** 10.1002/jcla.23924

**Published:** 2021-09-26

**Authors:** Peng Zhao, Xiaohong Zhao

**Affiliations:** ^1^ Department of Endocrinology and Rheumatology Immunology 3201 Hospital Hanzhong China

**Keywords:** clinical characteristics, disease risk, Long noncoding RNA PCAT1, multiple myeloma, prognosis

## Abstract

**Background:**

Long noncoding RNA PCAT1 (lnc‐PCAT1) involves in the proliferation and drug sensitivity of multiple myeloma (MM), while its prognostic role in MM patients is still obscure. This study aimed to explore the association of lnc‐PCAT1 with MM risk, clinical characteristics, treatment response, progression‐free survival (PFS), and overall survival (OS).

**Methods:**

A total of 83 symptomatic MM patients were enrolled in this study. Additionally, 30 healthy bone marrow donors as health controls were also recruited. Bone marrow plasma cell samples of MM patients and health donors were collected. Lnc‐PCAT1 in bone marrow plasma cells was detected by reverse transcription‐quantitative polymerase chain reaction.

**Results:**

Lnc‐PCAT1 was increased in MM patients than in health donors (*p *< 0.001), and receiver operating characteristic (ROC) curve showed that lnc‐PCAT1 had excellent capability of discriminating MM patients from health donors (area under curve: 0.932, 95% confidence interval: 0.889–0.976). In MM patients, lnc‐PCAT1 was correlated with bone lesion (*p *= 0.024), higher β_2_‐MG (*p *= 0.005), LDH (*p *= 0.037), and presence of Del (17p) (*p *= 0.029). Lnc‐PCAT1 was also associated with poor ISS stage (*p *= 0.013) and R‐ISS stage (*p *= 0.005). Besides, lnc‐PCAT1 was reduced after treatment (*p *< 0.001); meanwhile, lnc‐PCAT1 before treatment was correlated with lower CR (*p *= 0.046) but not ORR (*p *= 0.185). Additionally, lnc‐PCAT1 after treatment was associated with lower CR (*p *= 0.003) and ORR (*p *= 0.010). Furthermore, baseline Inc‐PCAT1 high and Inc‐PCAT1 increase after treatment were correlated with worse PFS and OS (all *p *< 0.05).

**Conclusion:**

Lnc‐PCAT1 dysregulation serves as a biomarker for diagnosis and prognosis for MM.

## Graphical abstract

Long noncoding RNA PCAT1 (lnc‐PCAT1) involves in the proliferation and drug sensitivity of multiple myeloma (MM), while its prognostic role in MM patients is still obscure. This study aimed to explore the association of lnc‐PCAT1 with MM risk, clinical characteristics, treatment response, progression‐free survival (PFS), and overall survival (OS). A total of 83 symptomatic MM patients were enrolled in this study. Additionally, 30 healthy bone marrow donors as health controls were also recruited. Bone marrow plasma cell samples of MM patients and health donors were collected. Lnc‐PCAT1 in bone marrow plasma cells was detected by reverse transcription‐quantitative polymerase chain reaction. Lnc‐PCAT1 was increased in MM patients than in health donors (*p *＜ 0.001), and receiver operating characteristic (ROC) curve showed that lnc‐PCAT1 had excellent capability of discriminating MM patients from health donors (area under curve: 0.932, 95% confidence interval: 0.889–0.976). In MM patients, lnc‐PCAT1 was correlated with bone lesion (*p *= 0.024), higher β_2_‐MG (*p *= 0.005), LDH (*p *= 0.037), and presence of Del (17p) (*p *= 0.029). Lnc‐PCAT1 was also associated with poor ISS stage (*p *= 0.013) and R‐ISS stage (*p *= 0.005). Besides, lnc‐PCAT1 was reduced after treatment (*P *＜ 0.001); meanwhile, lnc‐PCAT1 before treatment was correlated with lower CR (*p *= 0.046) but not ORR (*p *= 0.185). Additionally, lnc‐PCAT1 after treatment was associated with lower CR (*p *= 0.003) and ORR (*p *= 0.010). Furthermore, baseline Inc‐PCAT1 high and Inc‐PCAT1 increase after treatment were correlated with worse PFS and OS (all *p *＜ 0.05). Lnc‐PCAT1 dysregulation serves as a biomarker for diagnosis and prognosis for MM.

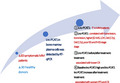

## 1 INTRODUCTION

Accounting for approximately 10% of hematologic malignancies, multiple myeloma (MM) is a heterogeneous disease characterized by uncontrolled growth of monoclonal plasma cells in the bone marrow.^1^, ^2^ Although there are several therapeutic methods for newly diagnosed MM, many patients still develop refractory or relapsed conditions, especially among elderly patients.^3^ Simultaneously, the 5‐year survival rate of MM disease is under 50%.^4^, ^5^ According to a previous report, there were estimated 16,500 new cases and 10,300 deaths of MM in China in 2016, while its mortality rate increased annually by 4.5% from 2006 to 2014.^6^ Therefore, it would be essential to find out more biomarkers for predicting the MM prognosis, which might strengthen the management of MM patients.

Long noncoding RNAs (lncRNAs) are transcripts with more than 200 nucleotides (nts) but with little protein‐coding ability.^7^ It is reported that some lncRNAs have participated in the progression of MM. For example, knockdown of lnc‐small nucleolar RNA host gene 16 suppresses MM cell proliferation by sponging miR‐342‐3p; lnc‐nuclear enriched abundant transcript 1 accelerates MM progression via the Janus kinase 2/signal transducer and activator of transcription 3 pathway; lnc‐NR_046683 highly expresses in drug‐resistant MM strains, which indicates that it is a potential drug target for MM treatment.

Among the commonly investigated lncRNAs, lncRNA prostate cancer‐associated transcript 1 (lnc‐PCAT1) involves in the progression of several malignancies.^8^ For example, overexpression of lnc‐PCAT1 expedites cell proliferation and migration in diffuse large B‐cell lymphoma through miR‐508‐3p/NFIB axis^9^; lnc‐PCAT1 promotes esophageal squamous cell proliferation by sponging miR‐326.^10^ Apart from these malignancies, lnc‐PCAT1 also participates in the progression of MM. For instance, a recent study shows that dysregulated lnc‐PCAT1 promotes proliferation in MM cells via p38 and jun N‐terminal kinase/mitogen‐activated protein kinase (JNK/MAPK) pathways.^11^ Additionally, enhanced lnc‐PCAT1 promotes plasma cell proliferation and inhibits apoptosis by downregulating microRNA‐129 (miR‐129) and further regulating mitogen‐activated protein kinase kinase kinase 7/nuclear factor‐kappaB (MAP3K7/NF‐κB) pathways in MM.^12^ Moreover, via modulating the protein kinase B/β‐catenin signaling pathway, lnc‐PCAT1 facilitates plasma cell proliferation, thus participating in the occurrence and progression of MM.^13^ Based on the above‐mentioned information, we hypothesized that lnc‐PCAT1 might be a potential biomarker for MM. However, no previous studies have been performed on this.

The present study was designed to investigate the association of lnc‐PCAT1 with MM risk and its clinical characteristics. Besides, this study also aimed to explore the correlation of lnc‐PCAT1 with MM prognosis, including treatment response, progression‐free survival (PFS), and overall survival (OS).

## 2 METHODS

### 2.1 Subjects

In this prospective study, 83 symptomatic MM patients admitted to the hospital between March 2017 and September 2020 were consecutively enrolled. All eligible patients were newly diagnosed as symptomatic MM according to the International Myeloma Working Group (IMWG) diagnostic criteria^14^ and had an age above 18 years old, willing to participate in the study and comply with study follow‐up. The patients were ineligible to be included in the study if they were identified as asymptomatic MM or had other malignant tumors. Meanwhile, pregnant patients were also excluded. Moreover, 30 healthy bone marrow donors were also recruited as health controls in the study analysis. Ethical approval for this study was obtained from the Institutional Review Board. Written informed consent was acquired from each recruited subject.

### 2.2 Lnc‐PCAT1 determination

For the investigation of lnc‐PCAT1 expression, bone marrow samples of MM patients were collected at diagnosis and at the completion of 3 to 4 cycles of induction therapy, respectively. The bone marrow samples of health donors were collected on their donation. Immediately after collection, CD138‐immunomagnetic beads (Miltenyi Biotec) were used to sort out plasma cells from the bone marrow samples. Subsequently, reverse transcription‐quantitative polymerase chain reaction (RT‐qPCR) was carried out to determine the expression of lnc‐PCAT1 in the plasma cells. The plasma cells were treated by TRIzol™ Reagent (Thermo Fisher Scientific) to extract total RNA, which were then submitted to perform reverse transcription using iScript™ cDNA Synthesis Kit (with random primer) (Bio‐Rad). After that, qPCR was carried out with QuantiNova SYBR Green PCR Kit (Qiagen). GAPDH was served as reference gene. The relative quantitative analysis of lnc‐PCAT1 expression was conducted with the use of 2^−ΔΔCt^ method. Primers were designed referring to a previous study.^12^

### 2.3 Data collection and assessment

Baseline clinical features and staging information (Durie‐Salmon (DS) stage, International Staging System (ISS) stage, and revised ISS (R‐ISS) stage^15^, ^16^, ^17^) of MM patients were documented after initial examinations. Induction therapy with regimen of lenalidomide/bortezomib/dexamethasone was administered for patients as recommended in the IMWG guideline.^18^ Response to induction therapy was evaluated at the completion of 3 to 4 cycles of induction therapy according to the IMWG criteria.^19^ For study analysis, patients with complete response (CR), very good partial response (VGPR), or partial response (PR) were recorded, and objective response rate (ORR) was calculated as the ratio of CR, VGPR, and PR patients in total patients. Patients without induction therapy response evaluation due to early death or loss of follow‐up were not included in the final analysis. Follow‐up and monitoring of patients were managed as recommended in the IMWG guideline.^18^ In the present study, the last follow‐up date was3/31/2021. Progression‐free survival (PFS) and overall survival (OS) were evaluated in accordance with IMWG guideline.^19^

### 2.4 Statistical analysis

SPSS 24.0 (IBM Corp.) and GraphPad Prism 7.01 (GraphPad Software Inc.) were used for data analysis and graph plotting, respectively. Categorical data were described as count with percentage. Continuous data distribution was analyzed using Kolmogorov–Smirnov (K–S) test, and median with interquartile range (IQR) or mean with standard deviation (SD) was calculated for descriptive analysis. Wilcoxon rank sum test or Kruskal–Wallis test was used for comparison of lnc‐PCAT1 expression in independent samples. Wilcoxon signed‐rank test was used for comparison of lnc‐PCAT1 expression in paired samples. The correlation of lnc‐PCAT1 with disease stage was analyzed by Spearman's rank correlation test. The receiver operating characteristic (ROC) curve and the area under the curve (AUC) were used to estimate the ability of lnc‐PCAT1 expression in identifying different subjects. Kaplan–Meier curve was plotted to display survival profiles of different subjects. Log‐rank test was applied for the determination of PFS and OS difference between subjects. In the survival analysis, “lnc‐PCAT1 decline” was defined as lnc‐PCAT1 expression declined after treatment compared with that before treatment; “lnc‐PCAT1 increase” was defined as lnc‐PCAT1 expression increased after treatment compared with that before treatment. Statistical significance was concluded if there was a *p* value < 0.05 in the corresponding analysis.

## 3 RESULTS

### 3.1 Baseline characteristics of MM patients

A total of 83 symptomatic MM patients were enrolled in this study. The baseline characteristics of MM patients were summarized in Table 1. In detail, the mean age of MM patients was 53.9 ± 8.5 years. There were 18 (21.7%) MM patients >60 years and 65 (78.3%) MM patients ≤60 years. The number of male patients was 51 (61.4%). Besides, the number of patients with bone lesion and with renal impairment was 62 (74.7%) and 33 (39.8%), respectively. Regarding immunoglobulin subtype, there were 47 (56.6%) patients with IgG, 15 (18.1%) patients with IgA, and 21 (25.3%) patients with other immunoglobulin subtypes. Besides, the mean values of hemoglobin and calcium were 98.5 ± 25.0 g/L and 9.9 ± 2.0 mg/dl, respectively. Meanwhile, the median values of serum creatinine, albumin, β_2_‐MG, and LDH were 1.8 (1.2–2.2) mg/dl, 34.0 (28.0–38.0) g/L, 5.6 (3.0–9.6) mg/L, and 206.3 (172.9–253.7) U/L, respectively. As for the chromosomal abnormality, there were 8 (9.6%) patients having t (4; 14), 4 (4.8%) patients having t (14; 16), and 6 (7.2%) patients having Del (17p). Additionally, 8 (9.6%) patients were at DS stage II and 75 (90.4%) patients were at DS stage III, while no patients were at DS stage I; 12 (14.5%) patients were at ISS stage I, 28 (33.7%) patients were at ISS stage II and 43 (51.8%) patients were at ISS stage III; 7 (8.4%) patients were at R‐ISS stage I, 38 (45.8%) patients were at R‐ISS stage II and 38 (45.8%) were at R‐ISS stage III.

**TABLE 1:** Baseline characteristics of MM patients

Items	MM patients (*N* = 83)
Age (years), mean ± SD	53.9 ± 8.5
>60 years, No. (%)	18 (21.7)
≤60 years, No. (%)	65 (78.3)
Male, No. (%)	51 (61.4)
Bone lesion, No. (%)	62 (74.7)
Renal impairment, No. (%)	33 (39.8)
Immunoglobulin subtype, No. (%)	
IgG	47 (56.6)
IgA	15 (18.1)
Others	21 (25.3)
Hemoglobin (g/L), mean ± SD	98.5 ± 25.0
Calcium (mg/dl), mean ± SD	9.9 ± 2.0
Serum creatinine (mg/dl), median (IQR)	1.8 (1.2–2.2)
Albumin (g/L), median (IQR)	34.0 (28.0–38.0)
β_2_‐MG (mg/L), median (IQR)	5.6 (3.0–9.6)
LDH (U/L), median (IQR)	206.3 (172.9–253.7)
Chromosomal abnormality, No. (%)	
t (4; 14)	8 (9.6)
t (14; 16)	4 (4.8)
Del (17p)	6 (7.2)
DS stage, No. (%)	
I	0 (0.0)
II	8 (9.6)
III	75 (90.4)
ISS stage, No. (%)	
I	12 (14.5)
II	28 (33.7)
III	43 (51.8)
R‐ISS stage, No. (%)	
I	7 (8.4)
II	38 (45.8)
III	38 (45.8)

Abbreviations: DS, Durie‐Salmon; IgA, immunoglobulin A; IgG, immunoglobulin G; IQR, interquartile range; ISS, International Staging System; LDH, lactate dehydrogenase; MM, multiple myeloma; R‐ISS, revised International Staging System; SD, standard deviation; β_2_‐MG, Beta‐2‐microglobulin.

### 3.2 Lnc‐PCAT1 expression in MM patients and health donors as well as its relation to MM risk

**FIGURE 1:**
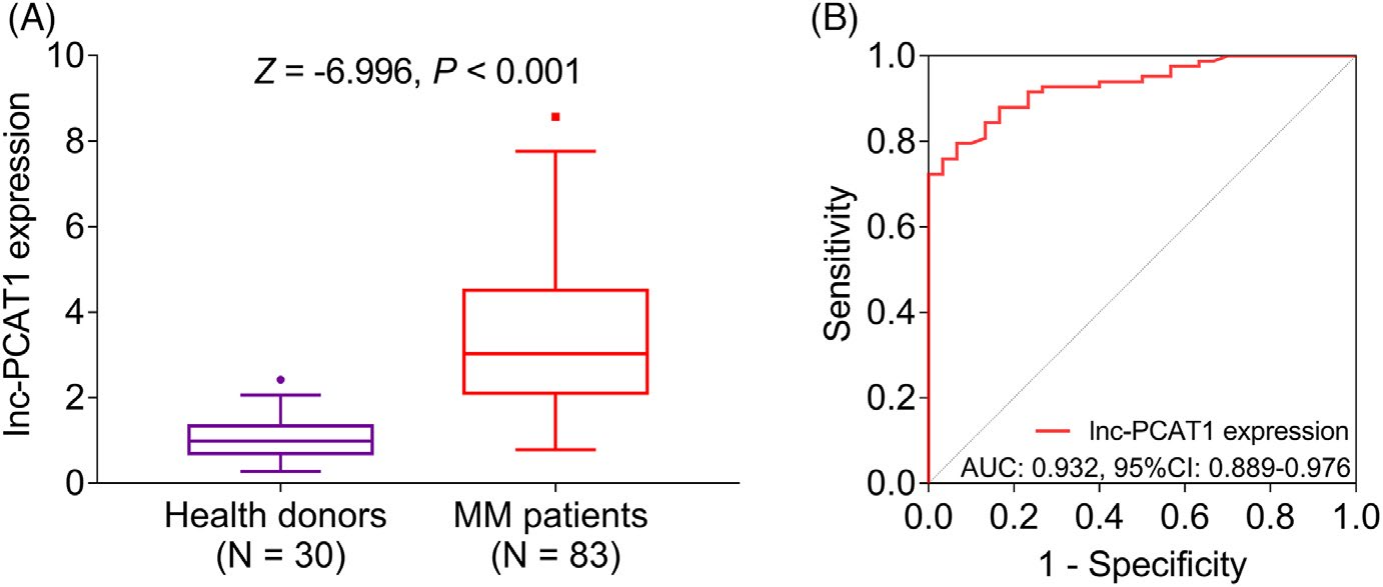
Lnc‐PCAT1 expression in MM patients. Comparison of lnc‐PCAT1 expression between MM patients and health donors (A); the capability of lnc‐PCAT1 expression to discriminate MM patients from health donors (B). AUC, area under curve; CI, confidence interval; lnc‐PCAT1, long noncoding RNA prostate cancer‐associated transcript 1; MM, multiple myeloma

Lnc‐PCAT1 expression was higher in the MM patients (*N* = 83) than in the health donors (*N* = 30) (*p *< 0.001) (Figure 1A). Besides, the ROC curve showed that lnc‐PCAT1 expression possessed excellent potential in discriminating MM patients from health donors with AUC of 0.932 (95% confidence interval (CI): 0.889–0.976) (Figure 1B).

### 3.3 Correlation of lnc‐PCAT1 with characteristics of MM patients

**FIGURE 2:**
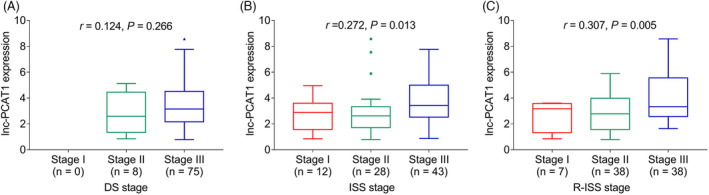
Association of lnc‐PCAT1 expression with MM stages. Correlation of lnc‐PCAT1 expression with DS stage (A), ISS stage (B), and R‐ISS stage (C). DS, Durie‑Salmon; ISS, International Staging System; lnc‐PCAT1, long noncoding RNA prostate cancer‐associated transcript 1; R‐ISS stage, Revised International Staging System

As shown in Table 2, lnc‐PCAT1 expression had positive association with bone lesion (*p *= 0.024), >5.5 mg/L β_2_‐MG (*p *= 0.005), >220 U/L LDH (*p *= 0.037), and presence of Del (17p) (*p *= 0.029) (Table 2). In addition, as suggested in Figure 2, lnc‐PCAT1 expression was correlated with elevated ISS stage (*p *= 0.013) (Figure 2B) and R‐ISS stage (*p *= 0.005) (Figure 2C). However, no correlations were observed between lnc‐PCAT1 expression and other characteristics of MM patients, including DS stage, age, gender, renal impairment, immunoglobulin subtype, hemoglobin, calcium, serum creatinine, albumin, and chromosomal abnormalities except Del (17p) (Figure 2A, Table 2) (all *p *> 0.05).

**TABLE 2:** Correlation between lnc‐PCAT1 and characteristics of MM patients

Items	lnc‐PCAT1 expression median (IQR)	*p* Value
Age		0.342
>60 years	3.009 (1.290–3.638)	
≤60 years	3.084 (2.214–4.639)	
Gender		0.392
Male	3.024 (2.514–4.535)	
Female	3.158 (1.636–4.118)	
Bone lesion		0.024
Yes	3.237 (2.429–4.709)	
No	2.580 (1.602–3.236)	
Renal impairment		0.270
Yes	3.242 (2.104–4.862)	
No	2.937 (2.069–3.831)	
Immunoglobulin subtype		0.184
IgG	3.148 (2.537–4.729)	
IgA	2.531 (1.643–3.416)	
Others	3.174 (1.598–4.304)	
Hemoglobin		0.623
>85 g/L	3.031 (2.164–4.703)	
≤85 g/L	3.114 (1.801–4.365)	
Calcium		0.802
>11.5 mg/dl	3.123 (1.773–4.106)	
≤11.5 mg/dl	3.031 (2.103–4.574)	
Serum creatinine		0.419
>2 mg/dl	3.426 (1.719–4.929)	
≤2 mg/dl	2.986 (2.103–3.840)	
Albumin		0.783
>35 g/L	3.237 (2.113–4.448)	
≤35 g/L	2.986 (2.103–4.514)	
β_2_‐MG		0.005
>5.5 mg/L	3.573 (2.536–5.008)	
≤5.5 mg/L	2.599 (1.677–3.392)	
LDH		0.037
>220 U/L	3.242 (2.531–5.387)	
≤220 U/L	2.974 (1.570–4.118)	
Chromosomal abnormality		
t (4; 14)		0.130
Positive	3.826 (2.775–4.680)	
Negative	2.986 (1.967–4.422)	
t (14; 16)		0.195
Positive	5.188 (2.662–7.536)	
Negative	3.024 (2.043–4.422)	
Del (17p)		0.029
Positive	5.903 (2.635–7.848)	
Negative	3.024 (2.005–4.211)	

Abbreviations: IgA, immunoglobulin A; IgG, immunoglobulin G; IQR, interquartile range; LDH, lactate dehydrogenase; lnc‐PCAT1, long noncoding RNA prostate cancer‐associated transcript 1; MM, multiple myeloma; β_2_‐MG, Beta‐2‐microglobulin.

### 3.4 Lnc‐PCAT1 expression after induction treatment in MM

**FIGURE 3:**
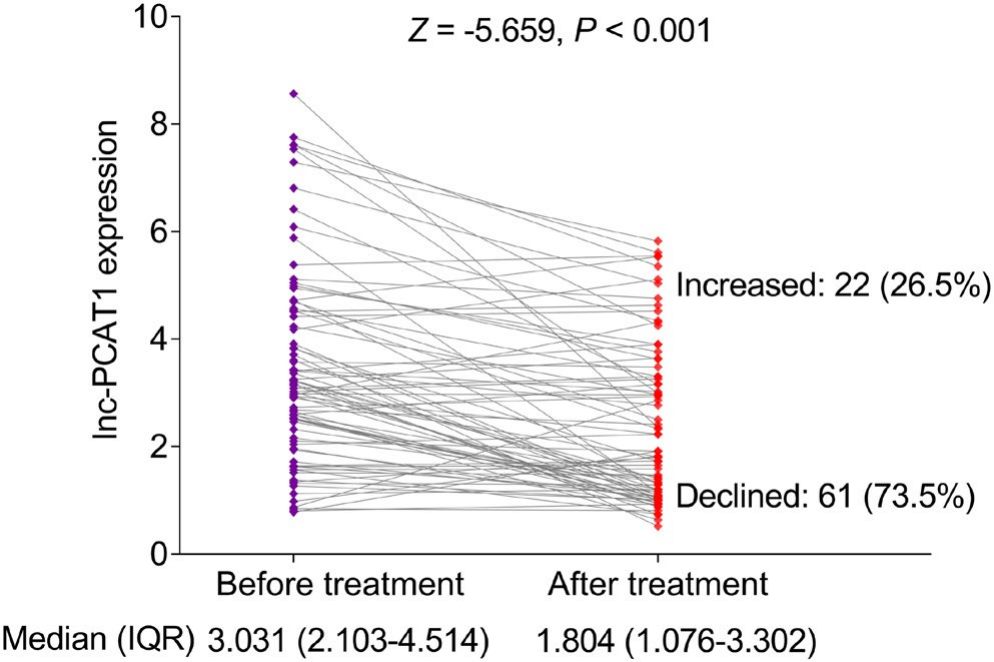
Change in lnc‐PCAT1 expression after treatment in MM. IQR, interquartile range; lnc‐PCAT1, long noncoding RNA prostate cancer‐associated transcript 1

**FIGURE 4:**
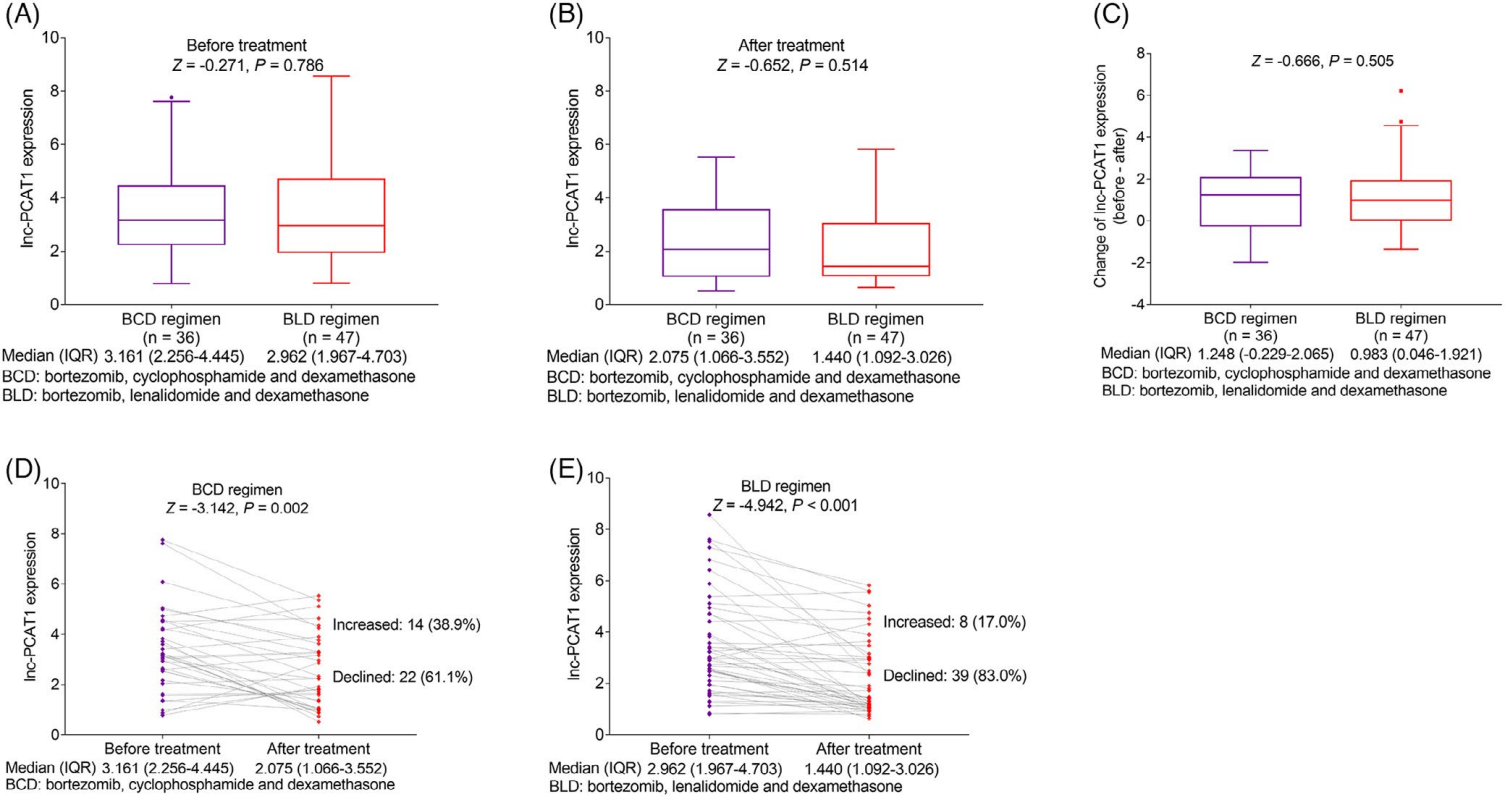
Comparison of lnc‐PCAT1 expression in MM patients received BCD and BLD regimens. Comparison of Lnc‐PCAT1 expression in MM patients received BCD and BLD regimens before treatment (A) and after treatment (B); comparison of change in lnc‐PCAT1 expression before treatment and after treatment in MM patients received BCD and BLD regimens (C); comparison of lnc‐PCAT1 expression in MM patients received BCD (D) and BLD regimens (E) after treatment. BCD, bortezomib, cyclophosphamide and dexamethasone; BLD, bortezomib, lenalidomide and dexamethasone; IQR, interquartile range; Lnc‐PCAT1, long noncoding RNA prostate cancer‐associated transcript 1

Lnc‐PCAT1 expression was decreased after treatment (median value: 1.804 (1.076–3.302)) compared with before treatment (median value: 3.031 (2.103–4.514)) (*p *< 0.001). Besides, lnc‐PCAT1 expression was declined in 61 (73.5%) patients and increased in 22 (26.5%) patients after treatment (Figure 3). In this study, MM patients mainly received bortezomib, cyclophosphamide, and dexamethasone (BCD) as well as bortezomib, lenalidomide, and dexamethasone (BLD) regimens as treatment methods. No difference was observed in lnc‐PCAT1 expression between patients received BCD and BLD before treatment or after treatment (both *p *> 0.05) (Figure 4A and B). Besides, no difference was observed in the change in lnc‐PCAT1 expression before and after treatment between MM patients received BCD and BLD (*p *> 0.05) (Figure 4C). However, in both patients received BCD (*p *= 0.002) and BLD regimens (*p *< 0.001), lnc‐PCAT1 expression was decreased after treatment (Figure 4D and E).

### 3.5 Correlation of lnc‐PCAT1 expression with treatment response in MM

**FIGURE 5:**
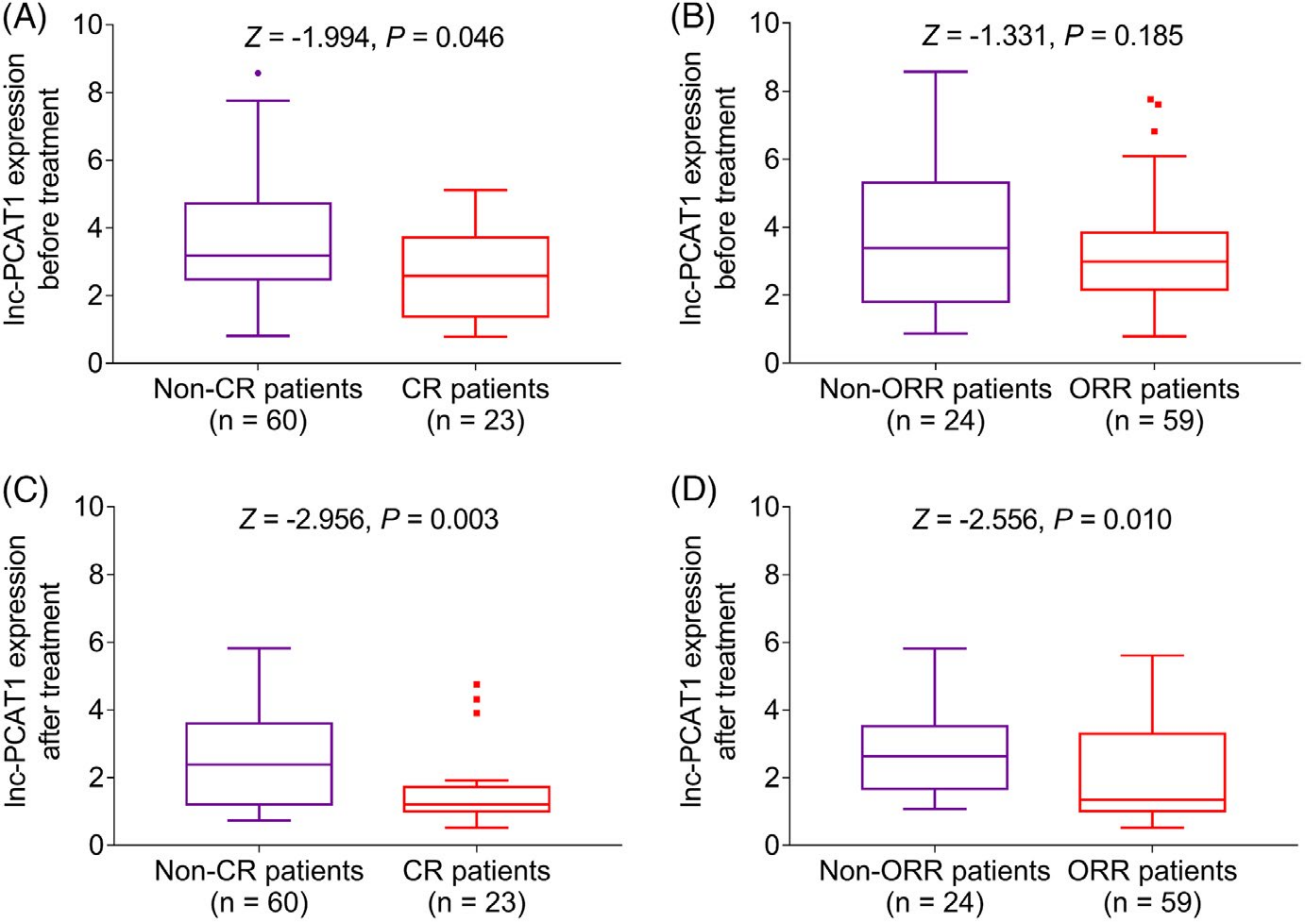
Association of lnc‐PCAT1 expression before/after treatment with CR and ORR in MM. Comparison of lnc‐PCAT1 expression before treatment between CR patients and non‐CR patients (A); comparison of lnc‐PCAT1 expression before treatment between ORR patients and non‐ORR patients (B); comparison of lnc‐PCAT1 expression after treatment between CR patients and non‐CR patients (C); Comparison of lnc‐PCAT1 expression after treatment between ORR patients and non‐ORR patients (D). CR, complete response; lnc‐PCAT1, long noncoding RNA prostate cancer‐associated transcript 1; ORR, objective response rate

**FIGURE 6:**
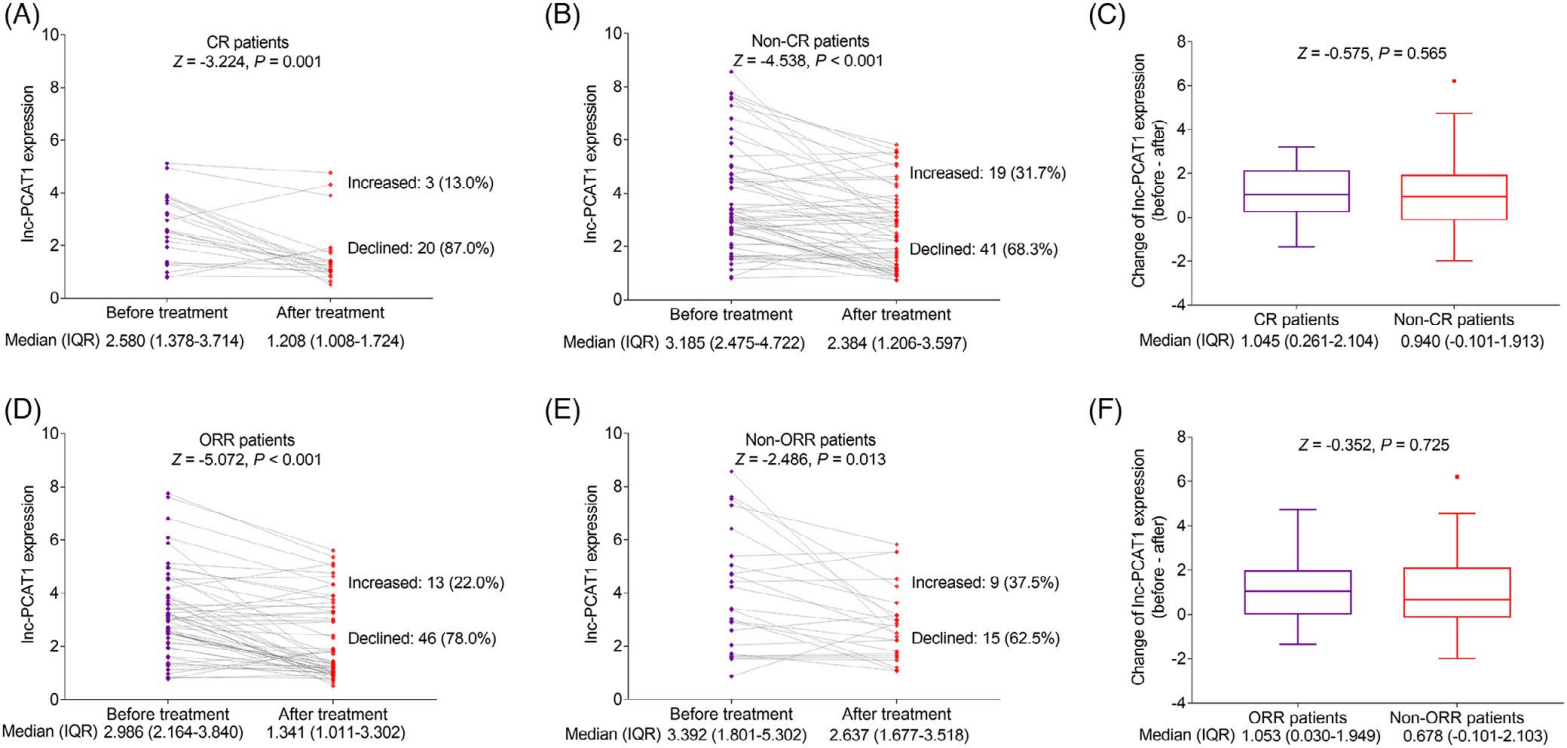
Change in lnc‐PCAT1 expression in CR, non‐CR, ORR, and non‐ORR patients in MM. Change in lnc‐PCAT1 expression in CR (A) and non‐CR (B) patients before and after treatment; comparison of change in lnc‐PCAT1 expression before and after treatment in CR and non‐CR patients (C); change in lnc‐PCAT1 expression in ORR (D) and non‐ORR (E) patients before and after treatment; comparison of change in lnc‐PCAT1 expression before and after treatment in ORR and non‐ORR patients (F). CR, complete response; IQR, interquartile range; lnc‐PCAT1, long noncoding RNA prostate cancer‐associated transcript 1; ORR, objective response rate

Lnc‐PCAT1 expression before treatment in the CR patients (*n* = 23) was lower than that in the non‐CR patients (*n* = 60) (*p *= 0.046) (Figure 5A). Otherwise, lnc‐PCAT1 expression before treatment was similar between the ORR patients (*n* = 59) and the non‐ORR patients (*n* = 24) (*p *= 0.185) (Figure 5B). In addition, lnc‐PCAT1 expression after treatment was reduced in the CR patients (*n* = 23) than the non‐CR patients (*n* = 60) (*p *= 0.003) (Figure 5C). Moreover, lnc‐PCAT1 expression after treatment was declined in the ORR patients (*n* = 59) compared with that in the non‐ORR patients (*n* = 24) (*p *= 0.010) (Figure 5D). Additionally, lnc‐PCAT1 expression showed a more predominant reduction in CR patients. In detail, lnc‐PCAT1 expression was greatly decreased in both CR patients (*p *= 0.010) (Figure 6A) and non‐CR patients (*p *< 0.001) (Figure 6B) after treatment compared with before treatment. No obvious difference was found in the change in lnc‐PCAT1 expression before and after treatment between CR and non‐CR patients (*p *> 0.05) (Figure 6C). In addition, lnc‐PCAT1 expression was declined in both ORR (*p *< 0.001) patients and non‐ORR patients (*p *= 0.013) after treatment compared with before treatment (Figure 6D and E). No obvious difference was found in the change in lnc‐PCAT1 expression before and after treatment between ORR and non‐ORR patients (Figure 6F) (*p *> 0.05).

### 3.6 Correlation of lnc‐PCAT1 expression with accumulating PFS and OS in MM

**FIGURE 7:**
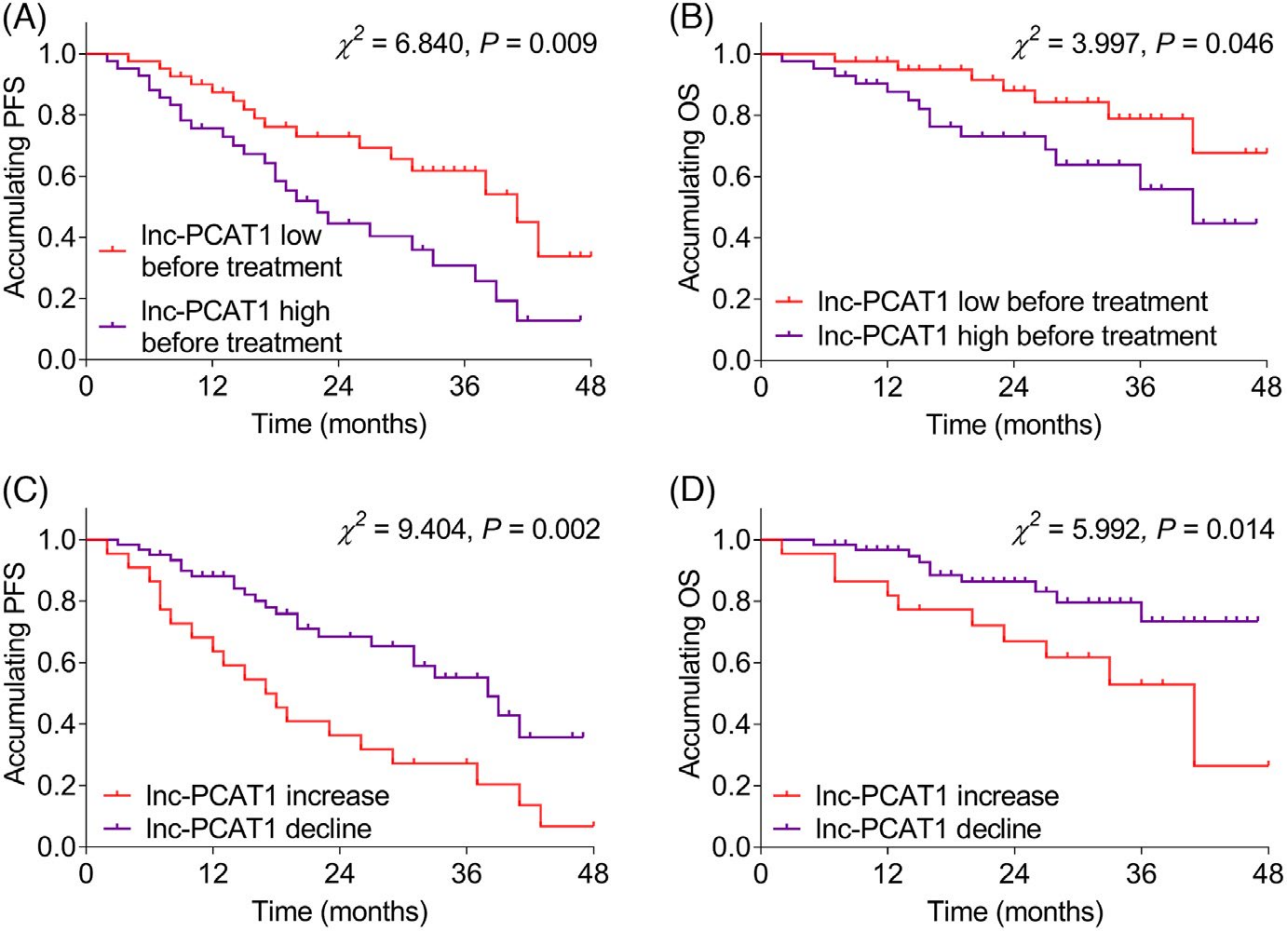
Association of lnc‐PCAT1 with accumulating PFS and OS. Association of baseline lnc‐PCAT1 high expression with accumulating PFS (A); association of baseline lnc‐PCAT1 high expression with accumulating OS (B); correlation of lnc‐PCAT1 increase after treatment with accumulating PFS (C); correlation of lnc‐PCAT1 increase after treatment with accumulating OS (D). lnc‐PCAT1, long noncoding RNA prostate cancer‐associated transcript 1; OS, overall survival; PFS, progression‐free survival

**FIGURE 8:**
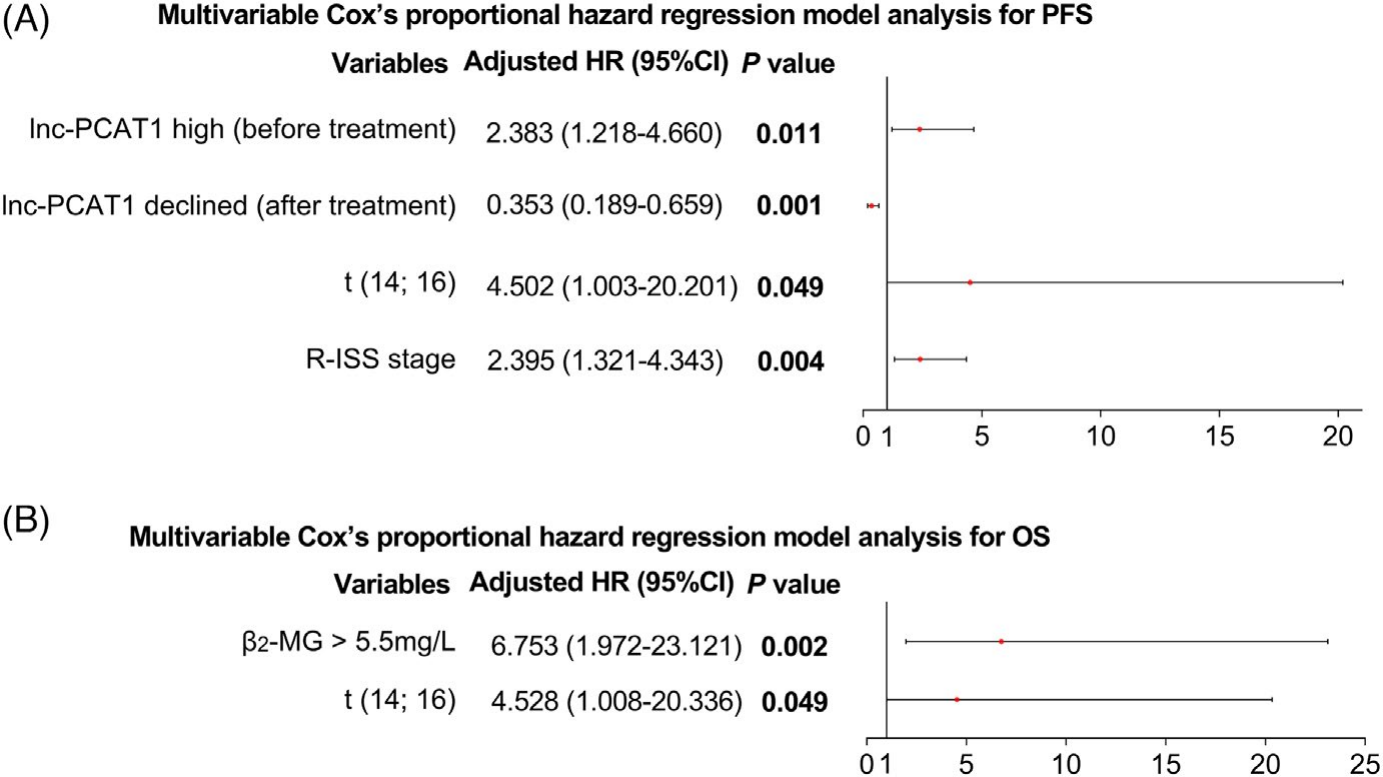
Independent prognostic factors for PFS and OS by multivariate Cox's analyses. Multivariable Cox's proportional hazard regression model analyses for PFS (A) and OS (B). CI, confidence interval; HR, hazard ratio; lnc‐PCAT1, long noncoding RNA prostate cancer‐associated transcript 1; OS, overall survival; PFS, progression‐free survival; R‐ISS, Revised International Staging System

Baseline lnc‐PCAT1 high expression (defined as lnc‐PCAT1 expression exceeded 3.031, which was the median value of lnc‐PCAT1 expression in MM patients before treatment) was correlated with shorter accumulating PFS (*p *= 0.009) (Figure 7A) and accumulating OS (*p *= 0.046) (Figure 7B). What is more, lnc‐PCAT1 increase (vs. lnc‐PCAT1 decline) after treatment was associated with worse accumulating PFS (*p *= 0.002) (Figure 7C) and accumulating OS (*p *= 0.014) (Figure 7D). In addition, by multivariable Cox's proportional hazard regression model analysis, lnc‐PCAT1 high (vs. low) (before treatment) (*p *= 0.011, HR (95% CI) = 2.383 (1.218–4.660)), t(14; 16) (*p *= 0.049, HR (95% CI) = 4.502 (1.003–20.201)), and R‐ISS stage (*p *= 0.004, HR (95% CI) = 2.395 (1.321–4.343)) were all independent predictive factors for shorter PFS; meanwhile, lnc‐PCAT1 decline (vs. increase) (after treatment) was an independent predictive factor for longer PFS (*p *= 0.001, HR (95% CI) = 0.353 (0.189–0.659)) (Figure 8A). Moreover, β_2_‐MG >5.5 mg/L (*p *= 0.002, HR (95% CI) = 6.753 (1.972–23.121)) and t(14; 16) (*p *= 0.049, HR (95% CI) = 4.528 (1.008–20.336)) were independent predictive factors for worse OS (Figure 8B).

## 4 DISCUSSION

In this study, we aimed to explore the correlation of lnc‐PCAT1 with MM risk, clinical characteristics, and prognosis, which observed that (1) lnc‐PCAT1 expression was increased in the MM patients; (2) lnc‐PCAT1 expression was correlated with bone lesion, higher β_2_‐MG, LDH, and presence of Del (17p) in MM; meanwhile, lnc‐PCAT1 expression was associated with poor prognostic risk stratification, including ISS stage and R‐ISS stage; and (3) baseline lnc‐PCAT1 high expression and its longitude increase during treatment were correlated with worse CR, PFS and OS.

As to lnc‐PCAT1 expression in the hematological malignancies, lnc‐PCAT1 is enhanced in acute myeloid leukemia patients.^9^ Our study found that lnc‐PCAT1 was increased in the MM patients than the health donors. A possible explanation could be that lnc‐PCAT1 promotes malignant cell growth via different pathways, including NF‐κB pathway, MAPK‐, and Wnt/β‐catenin signaling pathways, which influences the development and pathogenesis of MM.^11^, ^12^, ^20^, ^21^

In terms of the association of lncRNA expression with clinical characteristics of MM, an interesting study shows that lnc‐PCAT1 is positively correlated with β_2_‐MG concentration of MM.^22^ In our study, lnc‐PCAT1 was positively correlated with bone lesion, higher β_2_‐MG, LDH, and presence of Del (17p) in MM; moreover, it was associated with poor risk stratification in ISS stage and R‐ISS stage. The possible reasons could be that (1) lnc‐PCAT1 promotes plasma cell growth, resulting in MM progression,^12^ which might contribute to the bone lesion and develop some forms of renal impairment, then further cause higher β_2_‐MG and LDH concentration^23^, ^24^; (2) lnc‐PCAT1 was positively associated with higher β_2_‐MG (above‐mentioned), which was a feature of the poor ISS stage; thus, lnc‐PCAT1 was related to the poor ISS stage; meanwhile, lnc‐PCAT1 was positively associated with higher LDH, which exceeded the normal level of LDH constituting the most advanced subtype of R‐ISS stage, thus lnc‐PCAT1 was correlated with poor R‐ISS stage.

Regarding the correlation of lnc‐PCAT1 expression with prognosis in patients with MM, previous studies elucidate that high expression of NIMA‐related kinase 2, a target gene of lnc‐PCAT1, is related to poor prognosis and inferior survival in MM.^20^, ^25^ Previous studies also show that other lncRNAs can be served as potential biomarkers in MM. For instance, high lnc‐CCTA1 correlates with poor OS in MM patients and serves as a potential biomarker for the prognosis of MM patients^26^; in our study, we observed that baseline lnc‐PCAT1 high expression was correlated with worse CR, PFS, and OS. Possible reasons might be (1) according to a previous study, lnc‐PCAT1 expression inhibits bortezomib sensitivity in MM^11^; thus, it was related to unfavorable treatment response and further affected PFS and OS; (2) lnc‐PCAT1 expression was associated with poor risk stratification as mentioned above, including poor ISS stage and R‐ISS stage, which might indirectly result in worse PFS and OS. Our study also found that longitude increase in lnc‐PCAT1 during treatment had correlation with poor CR, PFS, and OS. The explanation might be that elevated lnc‐PCAT1 promotes plasma cell proliferation and inhibits apoptosis through downregulating miR‐129 and further modulating MAP3K7/NF‐κB pathways in MM.^12^ Therefore, its elevation after treatment might cause deteriorate MM disease condition, then resulting in poor treatment response and subsequently bringing in worse PFS and OS.

Although a lot of findings were identified in this study, there were still some limitations. First, with a small sample size, this study might have low statistical power. Further validation in the more and same number of patients and health controls might be needed. Second, our study evaluated the correlation of lnc‐PCAT1 expression with clinical characteristics and prognosis of patients having symptomatic MM, while the correlation of lnc‐PCAT1 expression with asymptomatic MM, might be further evaluated. Third, this study did not investigate the molecular mechanism of lnc‐PCAT1 involved in MM progression; thus, in vivo and in vitro experiments might be necessary to be further conducted.

Conclusively, lnc‐PCAT1 associates with elevated disease risk and unfavorable ISS stage, R‐ISS stage, treatment response, and survival of MM. It may potentially serve as a biomarker to predict MM prognosis, further improving the management of MM patients.

## CONFLICTS OF INTEREST

The authors declare that they have no conflicts of interest.

## Data Availability

Data sharing was not applicable to this article as no datasets were generated or analyzed during the current study.
